# Left atrial CT volume and CHA_2_DS_2_-VASc score predict early pulmonary vein stump thrombus after left upper lobectomy

**DOI:** 10.1038/s41598-023-32240-0

**Published:** 2023-03-27

**Authors:** Koji Takumi, Hiroaki Nagano, Kazuhiro Ueda, Tadashi Umehara, Takuya Tokunaga, Go Kamimura, Masami Sato, Ryota Nakanosono, Takashi Yoshiura

**Affiliations:** 1 Departments of Radiology, Kagoshima University Graduate School of Medical and Dental Sciences, 8-35-1 Sakuragaoka, Kagoshima City, 890-8544 Japan; 2 Departments of General Thoracic Surgery, Kagoshima University Graduate School of Medical and Dental Sciences, 8-35-1 Sakuragaoka, Kagoshima City, 890-8544 Japan

**Keywords:** Cardiology, Cardiovascular diseases, Respiratory tract diseases, Risk factors

## Abstract

The purpose of this study is to clarify the feasibility of left atrial (LA) volume measurement and CHA_2_DS_2_-VASc score for predicting the development of pulmonary vein (PV) stump thrombus after left upper lobectomy (LUL). The study population comprised 50 patients who underwent LUL for pulmonary lesions. All patients were evaluated for the development of PV stump thrombus at 7 days after LUL. LA volume was measured using preoperative CT and the CHA_2_DS_2_-VASc score was evaluated. LA volume and CHA_2_DS_2_-VASc score were compared between patients with and without the development of PV stump thrombus using the Mann–Whitney *U* test. Receiver–operating characteristic (ROC) curve analysis was performed to evaluate the accuracy of prediction of PV stump thrombus development. PV stump thrombus was detected in 17 (33.4%) of the 50 patients. LA volume was significantly greater in patients who developed PV stump thrombus than in those without thrombus (79.7 ± 19.4 vs. 66.6 ± 17.0 mL, *p* = 0.040). CHA_2_DS_2_-VASc score was significantly higher in patients with PV stump thrombosis than in those without thrombus (3.4 ± 1.5 vs. 2.5 ± 1.5, *p* = 0.039). Area under the ROC curve values for predicting PV stump thrombus were 0.679, 0.676, and 0.714 for LA volume, CHA_2_DS_2_-VASc score, and their combination, respectively. In conclusion, LA volume measured using preoperative CT and CHA_2_DS_2_-VASc score may help predict the development of PV stump thrombus after LUL.

## Introduction

Lung cancer is the most common cancer worldwide and is also the leading cause of cancer death^1^. Surgery is generally suitable for people with early-stage lung cancer^2,3^, and lobectomy is the most commonly performed surgical procedure. Several studies have reported that pulmonary vein (PV) stump thrombus is a frequent occurrence after surgical treatment for lung cancer, especially left upper lobectomy (LUL)^4–8^, and the reported incidence of PV stump thrombus after LUL ranges from 13.5 to 17.9%^4,5^. In addition, PV stump thrombus after LUL can cause postoperative cerebral infarction^7,9^. Therefore, there is growing recognition of the importance of the pathogenesis and effective prevention of PV stump thrombus after LUL. Recent studies have suggested that postoperative blood flow, especially blood stasis and turbulent blood flow in the PV stump, contributes to the development of thrombus^10–12^. The potential role of a longer stump of the left superior PV in PV stump thrombosis has been suggested, which can provide a procoagulant environment such as turbulent flow or blood stasis^13^. However, PV stump thrombus after LUL still occurs in patients who undergo proximal ligation of the PV stump after LUL^14,15^.

The left atrial (LA) appendage has a structure similar to that of the PV stump after LUL, which is connected to the LA and has a blind end. LA appendage thrombus is commonly seen in patients with atrial fibrillation (AF) and is a risk factor for stroke. LA appendage thrombus has been reported to be associated with LA volume measured using contrast-enhanced CT^16^ and LA enlargement has been reported to be associated with an increased risk of stroke and systemic embolization^17–19^. The usefulness of CHA_2_DS_2_-VASc score (congestive heart failure, hypertension, age ≥ 75 years, diabetes mellitus, stroke or transient ischemic attack, vascular disease, age 65 to 74 years, and sex category) has also been widely demonstrated for predicting LA thrombus and stroke risk in patients with atrial fibrillation^20^. Therefore, we hypothesized that LA volume measured using CT images and CHA_2_DS_2_-VASc score would be useful for predicting PV stump thrombus after LUL. The purpose of this study was to clarify the feasibility of LA volume measurement and CHA_2_DS_2_-VASc score for predicting the development of PV stump thrombus after LUL.

## Methods

This study was approved and the requirement for informed consent from the study subjects was waived by the institutional review board (Ethics Committee on Epidemiological and its related Studies, Sakuragaoka Campus, Kagoshima University; approval number, 220,009) due to the retrospective study design. This study was conducted in accordance with the Declaration of Helsinki and Ethical Guidelines for Medical and Health Research Involving Human Subjects in Japan.

### Patients

A retrospective review of the CT imaging database and clinical records of our radiology department identified 936 consecutive patients with suspected lung neoplasms who had undergone pretreatment chest CT examination between January 2018 and October 2020. Among these, 50 patients (27 men and 23 women; mean age, 68 ± 10 years; age range, 41–83 years) met the following inclusion criteria and were included in this study: (1) had undergone LUL; (2) the presence or absence of PV stump thrombus had been evaluated at 7 days after lobectomy using cine-MR images with or without contrast-enhanced cardiac CT.

### Preoperative CT examination and left atrial volume measurement

CT examinations were obtained on a 64-multidetector row CT scanner (IQon spectral CT, Philips Healthcare). The CT scan parameters were as follows: tube voltage, 120 kVp; effective tube current–time product, 160 mAs with automatic tube current modulation; rotation time, 0.4 s; pitch, 0.703; and collimator configuration, 64 × 0.625 mm. Scan timing was initiated 50 s after attenuation in the ascending aorta increased to a default threshold (150 HU), as measured by a dedicated monitoring system after intravenous administration of a nonionic contrast agent (Omnipaque 300 mgI/mL, Daiichi Sankyo, Tokyo, Japan). The injected contrast agent dose was 2.0 mL/kg body weight, up to a maximum dose of 150 mL for patients weighing ≥ 75.0 kg, and injection time was fixed at 30 s.

All CT images were transferred and analyzed using a thin-client workstation (SYNAPSE VINCENT, Fujifilm Medical Co., Tokyo, Japan). LA volume was measured by a radiologist with 20 years of chest radiology experience (K.T.) who was blinded to the final results of the presence or absence of PV thrombus after surgery. The 3D volume of the LA was semiautomatically obtained and measured using an automated analysis program installed in the workstation.

### ***Evaluation of CHA***_***2***_***DS***_***2***_***-VASc score***

The CHA_2_DS_2_-VASc score was calculated for each patient as follows: two points were assigned for a history of stroke or transient ischemic attack (TIA), or age > 75 years; and 1 point was assigned for age 65–74 years, history of hypertension, diabetes mellitus, cardiac failure, vascular disease (myocardial infarction, complex aortic plaque and peripheral arterial disease) and female sex^21^. Heart failure was defined as clinical heart failure (any history of systolic heart failure or ejection fraction < 40%). Hypertension was defined as high blood pressure (> 140/90 mm Hg) or receiving treatment with antihypertensive drugs. Diabetes mellitus was defined as a fasting plasma glucose level > 126 mg/dl or the use of oral anti-diabetic drugs and/or insulin. Vascular disease was defined as intermittent claudication, amputation, lower extremity revascularization, history of myocardial infarction, or complex aortic plaques on CT imaging.

### Evaluation of PV stump thrombus after LUL

Since November 2017, cine-MR imaging has been a part of our institute’s routine clinical protocol for evaluating the development of PV stump thrombus after LUL. Cardiac cine-MR imaging is considered a reliable diagnostic method for the evaluation of thrombus in the LA and LA appendage^22^. All patients had undergone cine-MR examinations on postoperative day 7 using a 3 T system (Prisma, Siemens Healthcare, Erlangen, Germany) with a 30-channel body array coil. Cine images were acquired in the coronal plane using the balanced steady state free precession (bSSFP) sequence (repetition time, 40–80 ms; echo time, 1.1 ms; flip angle, 48°; number of cardiac phases, 10–15; number of signals averaged, 1; field-of-view, 360 × 360 mm^2^; in-plane spatial resolution 1.9 × 1.9 mm; section thickness, 5 mm; number of slices, 27) with short periods of breath-holding. Thrombus was defined as a mass within the PV stump or LA cavity that had margins distinct from the PV stump or LA wall, and distinguishable from technical or flow artifact. All cases of suspected thrombus on cine-MR imaging underwent contrast-enhanced ECG-gated cardiac CT to confirm the presence of thrombus. The size of the PV stump thrombus was also measured in the axial plane on cardiac CT.

### Statistical analysis

LA volume and CHA_2_DS_2_-VASc score were compared between patients with and without the development of PV stump thrombus, using the Mann–Whitney *U* test. Spearman’s rank correlation coefficient was used to measure the association between LA volume and CHA_2_DS_2_-VASc score. Receiver–operating characteristic (ROC) curve analysis was performed to evaluate the accuracy of predicting the development of PV stump thrombus, for each of LA volume, CHA_2_DS_2_-VASc score, and their combination. The combination was considered positive when both parameters were positive. For each parameter, optimal cutoff values were chosen using a threshold criterion that maximized the Youden index for predicting PV stump thrombus. The area under the ROC curve (AUC) for LA volume, CHA_2_DS_2_-VASc score, and their combination were compared using DeLong’s test^23^. All data for continuous variables are presented as the mean ± standard deviation (SD). A *P-*value < 0.05 was considered to indicate statistical significance in all analyzes. Statistical analyses were performed using MedCalc version 20.1 (MedCalc Software, Mariakerke, Belgium) and SPSS version 28.0 (SPSS, Chicago, IL).

## Results

Table 1 summarizes patients’ clinical characteristics according to the presence or absence of PV stump thrombosis after LUL. PV stump thrombus was detected in 17 (33.4%) of the 50 patients who underwent LUL (9 men and 8 women; mean age, 70 ± 9 years; age range, 45–83 years). Mean thrombus size was 12.5 ± 3.8 mm. All patients were successfully treated with direct oral anticoagulants (8 patients treated with edoxaban, 4 with rivaroxaban, and 2 with apixaban) or a combination of heparin and warfarin (3 patients) without systemic thromboembolism. All patients with PV stump thrombus underwent follow-up contrast-enhanced CT within 7–60 days after treatment and confirmed improvement.

**Table 1:** Clinical characteristics according to PV stump thrombosis formation after left upper lobectomy.

Variables	Thrombus (n = 17)	Non-thrombus (n = 33)	*p*
Gender (M: F)	9: 8	18 : 15	1.000
Age (years)	70 ± 9	67 ± 11	0.315
Tumor types (AD/SCC/Metastasis)	11/3/3	25/6/2	0.428
Preoperative medications			
Antihypertensive drugs	47.1% (8/17)	33.3% (11/33)	0.373
Anticoagulant or antiplatelet drugs	11.8% (2/17)	6.1% (2/33)	0.597
Steroid drugs	11.8% (2/17)	0% (0/33)	0.111
Atrial fibrillation	5.9% (1/17)	0% (0/33)	0.340
Hypertension	52.9% (9/17)	33.3% (11/33)	0.229
Diabetes Mellitus	17.6% (3/17)	12.1% (4/33)	0.677
Congestive heart failure	0% (0/17)	0% (0/33)	-
Vascular disease	76.5% (13/17)	54.5% (18/33)	0.218
Stroke	11.8% (2/17)	0% (0/33)	0.111
CHA_2_DS_2_-VASc score	3.4 ± 1.5	2.5 ± 1.5	**0.039**
Left atrium volume (mL)	79.7 ± 19.4	66.6 ± 17.0	**0.040**

AD, adenocarcinoma; SCC, squamous cell carcinoma .

Continuous values are presented as mean ± SD.

Significant values are in bold.

**Figure 1:**
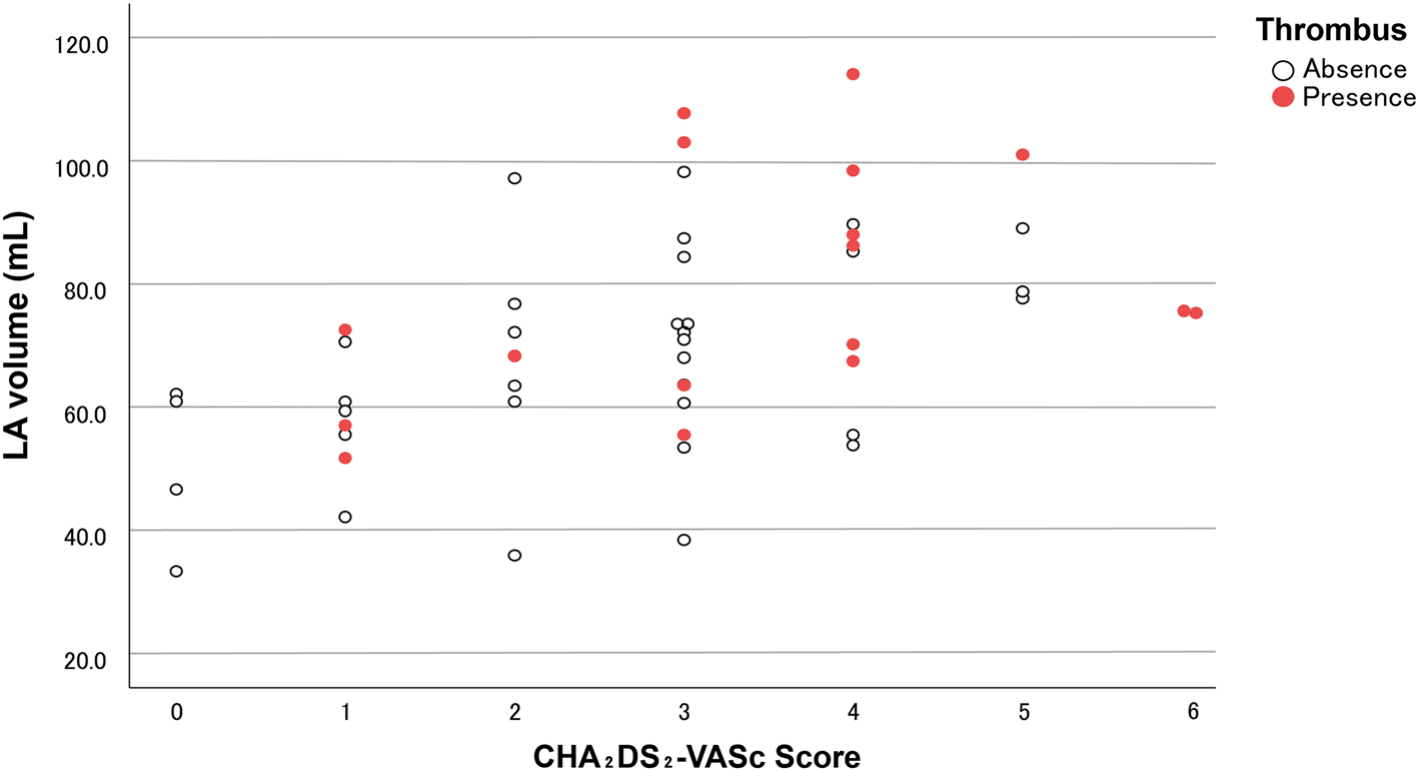
Scatterplot of left atrial volume versus CHA_2_DS_2_-VASc score. Patients who developed pulmonary vein stump thrombus tended to be clustered in the area of large left atrial volume and high CHA_2_DS_2_-VASc score.

LA volume was significantly greater in patients with development of PV stump thrombus (79.7 ± 19.4 mL) than in those without thrombus (66.6 ± 17.0 mL, *p* = 0.040). CHA_2_DS_2_-VASc score was significantly higher in patients with PV stump thrombosis (3.4 ± 1.5) compared with those without thrombus (2.5 ± 1.5, *p* = 0.039). LA volume was significantly correlated with CHA_2_DS_2_-VASc score (ρ = 0.538, *p* < 0.001). The LA volume and CHA_2_DS_2_-VASc score of each patient are plotted in Fig. 1. There was a trend for cases of development of PV stump thrombus to cluster in the area of large LA volume and high CHA_2_DS_2_-VASc score.

**Figure 2:**
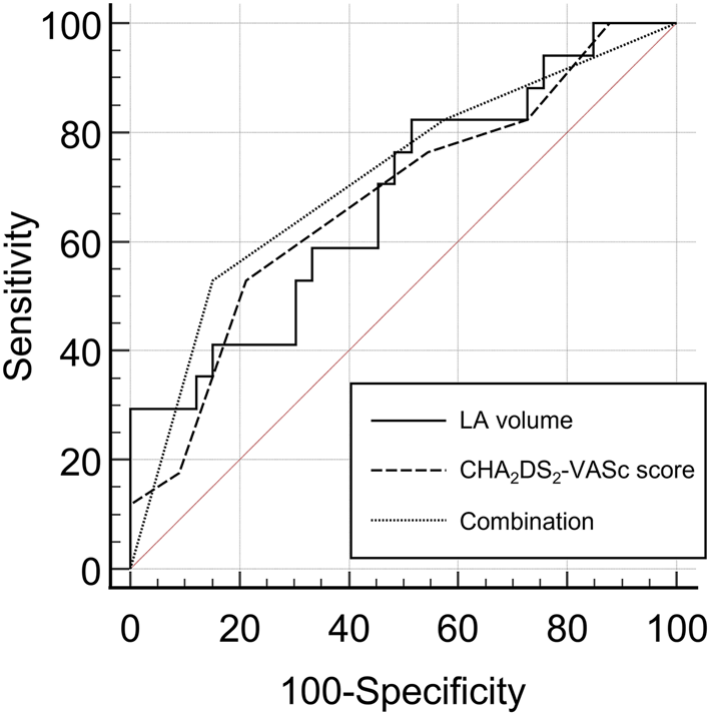
Receiver–operating characteristic (ROC) curve analysis for predicting pulmonary vein stump thrombus after left upper lobectomy. The area under the ROC curve values were 0.679, 0.676, and 0.714 for left atrial volume, CHA_2_DS_2_-VASc score, and the combination of the two parameters, respectively.

For cutoff values of 63.48 mL for LA volume and ≥ 4 for CHA_2_DS_2_-VASc score, the AUC values for predicting the development of PV stump thrombus were 0.679 (95% confidence interval (CI), 0.532–0.804) and 0.676 (95%CI, 0.528–0.801) for LA volume and CHA_2_DS_2_-VASc score, respectively. AUC for the combination of the two parameters was 0.714 (95%CI, 0.569–0.833) (Fig. 2), which showed no significant difference compared to each parameter alone (all > 0.05).

**Figure 3:**
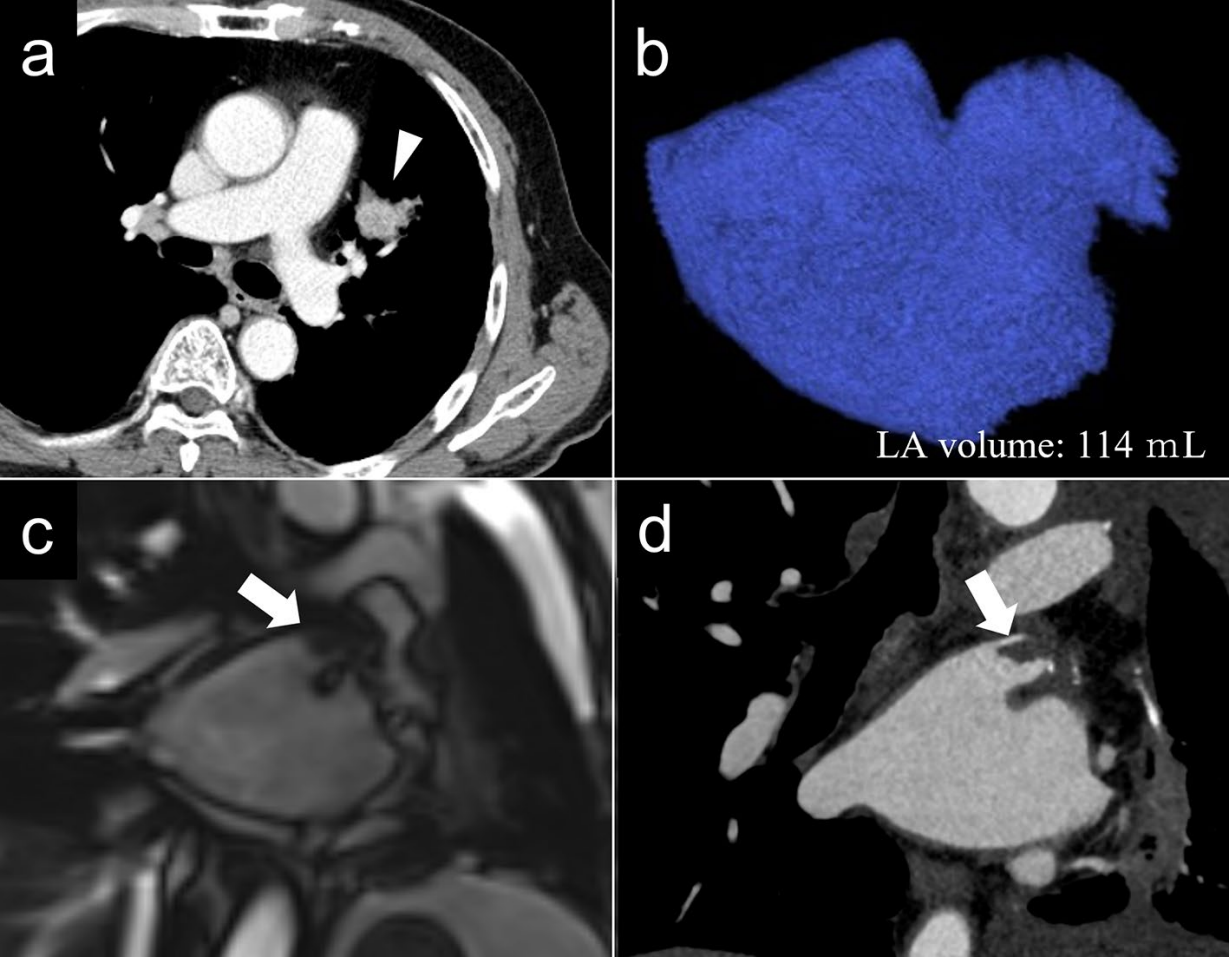
A 73-year-old man with lung cancer who underwent left upper lobectomy. Chest CT (**a**) reveals a nodule (arrowhead) in the left upper lobe. 3D-volume-rendered image (**b**) shows left atrial volume of 114 mL. The CHA_2_DS_2_-VASc score was 4. Postoperative cine-MRI (**c**) and contrast-enhanced cardiac CT (**d**) reveal the left superior pulmonary vein stump thrombus (arrows).

Figure 3 shows a representative case of PV stump thrombus.

## Discussion

This study focused on the potential of LA volume and CHA_2_DS_2_-VASc score to predict the development of PV stump thrombus after LUL. Our results suggest that larger LA volume and higher CHA_2_DS_2_-VASc score are associated with the development of PV stump thrombus after LUL. LA volume measurement can be calculated using contrast-enhanced CT, which is commonly performed for preoperative evaluation of lung cancer; and CHA_2_DS_2_-VASc score can be easily calculated for almost all patients using their clinical information. Thus, our approach is readily applicable to clinical practice.

LA enlargement has been reported to be associated with increased incidence of cardiovascular events such as stroke, congestive heart failure, cardiovascular death, and atrial fibrillation^19^. Several studies that included echocardiography have revealed a strong relationship between LA dilatation and LA appendage thrombus^24,25^. LA enlargement can promote blood stasis, which facilitates thrombus formation in the LA appendage^26^. Similarly, PV stump thrombus formation after lobectomy may be affected by blood stasis associated with LA dilation. PV stump thrombus has been reported to occur frequently in patients treated with LUL^5–8^. Some characteristic regional hemodynamics in the LA have been observed more commonly after LUL than after other lung lobectomies, and blood turbulence around the PV stump could be associated with thrombus formation after LUL. Ohtaka et al. reported that slow blood flow and the presence of spontaneous echo contrast in the left superior PV stump were associated with PV stump thrombus^10^. Recently reported 4D flow MRI analyses have revealed that LUL likely causes blood turbulence near the vein stump by complicated blood streams in the LA, which can play a part in developing vein stump thrombus^11,12^. PV stump thrombus may be further promoted when blood flow stasis due to LA dilation is added to the blood flow dynamics after LUL.

The CHA_2_DS_2_-VASc score is an easily used tool for predicting stroke risk in patients with atrial fibrillation^20^ and has recently been demonstrated to play a predictive role for systemic thromboembolism and mortality in patients with sinus rhythm and no history of atrial fibrillation^27,28^. In the present study, CHA_2_DS_2_-VASc score showed a significant correlation with LA volume, which is consistent with the findings of a previous report^29^. In addition to LA volume, CHA_2_DS_2_-VASc score may be associated with the condition of blood flow in the LA. CHA_2_DS_2_-VASc score has been reported to be associated with low velocity in the LA appendage, and higher CHA_2_DS_2_-VASc score was considered to be a risk factor for LA appendage thrombus^30,31^. Furthermore, it has been reported that CHADS_2_ is correlated with the amount of LA fibrosis, which independently predicted cerebrovascular events^32^, and is also associated with LA function^33^. LA dysfunction can cause blood flow stasis and may result in the development of PV stump thrombus after lobectomy.

In the present study, the incidence of PV thrombus after LUL was 33.4% (17/50), which is slightly higher than previously reported^5–8^. This discrepancy may be attributable to differences in the timing of postoperative evaluation for PV thrombus. All of the present patients underwent cine MR examination on day 7 after surgery to assess PV stump thrombus. In contrast, previous studies identified PV stump thrombosis retrospectively by ultrasound or CT a few months after lobectomy. These studies may have overlooked some fraction of early asymptomatic thrombi that resolved spontaneously before the imaging evaluation, in turn underestimating the incidence of PV thrombosis after LUL. Thus, it is conceivable that our results more accurately indicate the frequency of PV stump thrombus development after LUL. Clinicians and radiologists should recognize that the incidence of PV stump thrombus might be higher than previously reported. Transesophageal echocardiography is considered the gold standard for detecting LA or LA appendage thrombus^34^. However, transesophageal echocardiography requires invasive procedures such as esophageal intubation and sedation. Contrast-enhanced cardiac CT imaging can detect LA thrombus with high accuracy, but requires the injection of contrast material, and thus it cannot be performed in patients with renal dysfunction and an allergic history. As an alternative non-invasive approach, cardiac MR imaging, including cine-MR, is a reliable diagnostic method for evaluation of thrombus in the LA and LA appendage^22,35,36^. Cine-MR imaging can also be applied to the evaluation of PV stump thrombus.

There are some limitations in the present study. First, there were fewer patients with PV stump thrombus than those without. Second, we used non-ECG-gated CT to measure LA volume. Although measurement is more accurate using ECG-gated CT, clinical CT for lung tumors is usually performed without ECG gating. A recent study^37^ has shown that non-ECG gated CT is a reliable method for measuring left and right atrial volumes in patients with normal atrial function, and is comparable to transthoracic echocardiography in terms of accuracy and reproducibility. Additionally, non-ECG gated CT can identify cases of LA enlargement with high specificity (94%)^38^. However, the significant difference between LA end-systolic and end-diastolic volumes highlights the need for ECG-gated imaging to accurately measure LA volume. Further investigations using ECG-gated CT or MRI are desired for a more accurate assessment of LA enlargement and its relationship with PV stump thrombus development. Finally, this study was conducted only in patients without late-stage CKD who have difficulty with contrast-enhanced CT examination. Cardiac dysfunction and renal failure are often closely related, therefore, PV stump thrombus may occur more frequently in late-stage CKD patients. Further study including late-stage CKD patients is desired. Nevertheless, this is the first study to focus on the feasibility of LA volume and CHA_2_DS_2_-VASc score to predict PV stump thrombosis after LUL. We believe that our pilot study encourages future research including a large multicenter prospective study.

In conclusion, the present pilot study demonstrated that greater LA volume and higher CHA_2_DS_2_-VASc score were significantly associated with PV stump thrombosis after LUL. The use of combined LA volume measurement using preoperative CT and CHA_2_DS_2_-VASc score may help predict the development of PV stump thrombus after LUL.

## Data Availability

The datasets generated and/or analyzed during this study are available from the corresponding author upon reasonable request.
